# Empathy and Schizotypy: A Network Comparison of the Interpersonal Reactivity Index in High and Low Schizotypy Groups

**DOI:** 10.3390/bs14030245

**Published:** 2024-03-18

**Authors:** Lillian A. Hammer, Aleksandr Karnick, Kendall Beals, Lauren Luther, Kelsey A. Bonfils

**Affiliations:** 1 Department of Psychology, University of Southern Mississippi, Hattiesburg, MS 39406, USA; aleksandrs.karnick@usm.edu (A.K.); kendall.beals@usm.edu (K.B.); kelsey.bonfils@usm.edu (K.A.B.); 2 Department of Psychiatry and Human Behavior, Brown University, Providence, RI 02912, USA; 3 Department of Psychology, University of Georgia, Athens, GA 30602, USA

**Keywords:** schizotypy, empathy, network analysis, network comparison, social cognition, interpersonal reactivity index, schizophrenia spectrum

## Abstract

Empathy is a multifaceted concept that is vital to effective social functioning; yet, it is impaired in high schizotypy groups. Furthermore, empathy has been found to be a mediator in the relationship between schizotypy and social functioning, highlighting the importance of empathy as a driver in social outcomes. Despite this, the four-factor structure of a widely-used measure of empathy—the Interpersonal Reactivity Index (IRI)—has been found to be psychometrically weak in high schizotypy samples. As such, this study aimed to assess differences in the item-level network of the IRI between high (*n* = 427) and low schizotypy groups (*n* = 470). The results reveal that there are significant differences in the structure of these networks, though they evidence similar strengths. Within the high schizotypy group, the network structure was consistent with the four-factor structure of the IRI subscales; items from each subscale clustered together and were distinct from those in the other subscales. By contrast, the low schizotypy group evidenced six clusters that did not mirror the IRI subscales. These results suggest that the item-level structure of the IRI is dependent upon the level of schizotypy of the sample, with the high schizotypy group’s network functioning similarly to what would be expected from the original four-factor structure.

## 1. Introduction

Empathy is a complex construct that includes our ability to share in the emotions of others (i.e., affective empathy) and understand others’ perspectives (i.e., cognitive empathy [1,2]). These facets of empathy have been shown to be related, but distinct from one another [2,3]. While some disagreement exists in the most effective way to measure empathy [4,5,6,7], one of the most widely used metrics is a self-report measure of four factors of empathy: the Interpersonal Reactivity Index (IRI [1]). The IRI includes the empathic concern subscale (IRI-EC), which measures one’s ability to feel sympathetic emotions towards others; the personal distress subscale (IRI-PD), which measures feelings of distress when exposed to other’s emotionally heightened states; the perspective-taking subscale (IRI-PT), which measures one’s ability to take the perspective of others; and the fantasy subscale (IRI-FS), which measures one’s ability to imagine themselves in the scenarios of characters in fictional stories, such as television or books [1,8]. The IRI has been used in numerous studies [9] that cross numerous countries and populations [10,11,12,13,14]. There have also been many examinations of the IRI’s psychometric properties that have questioned the optimal structure of this measure [9,15,16]. Appropriate assessment of empathy is vital, as it is closely linked to important outcomes, including social functioning [2]. Indeed, having higher levels of empathy are associated with positive social behaviors, such as forgiving others and behaving altruistically [2,17,18,19,20,21,22]. This underscores the importance of effective empathy measurement, particularly for those who might have poor social outcomes.

Deficits in social functioning and processes needed to guide functioning, including empathy, are prominent among people with schizophrenia spectrum disorders [23,24,25,26,27,28], those at clinical high risk for schizophrenia [29,30], and those with subclinical manifestations of schizophrenia-like symptoms [31,32,33]. One such manifestation is schizotypy, which encapsulates personality traits similar to subclinical symptoms of schizophrenia (e.g., lower reported interest in social relationships and higher levels of magical beliefs) and, at high levels, is linked to risk for the development of schizophrenia spectrum disorders [34,35]. Participants with high schizotypy, defined as those with scores in the top 5–10% of responses [35,36], have been consistently found to have lower empathy than participants with average or low levels of schizotypy traits [31,32,33]. Moreover, one study found that empathy mediated the relationship between schizotypy and social functioning, suggesting that empathy may be a primary driver in the negative impacts of schizotypy on social outcomes [31]. Therefore, understanding empathy, its facets, and its measurement in high schizotypy groups is imperative to furthering our understanding and ability to intervene on social outcomes for these individuals.

Previous research has begun to assess the structure of the IRI within a high schizotypy group. Specifically, Bonfils and colleagues [15] conducted a factor analysis on the IRI to determine whether the four-factor structure had adequate fit in a sample of high schizotypy participants. They found that the original four-factor structure did not evidence adequate fit in this group; by contrast, a two-factor model, including the perspective-taking and empathic concern subscales, provided the best fit [15]. This suggests that the IRI may vary in terms of how it performs in those with and without high levels of schizotypy. However, more work is needed to understand the inter-relations amongst empathy facets between these groups. This study takes a novel approach to further our understanding of the measurement of empathy and its facets in those with high and low schizotypy via item-level network analysis.

In contrast to the evaluation of psychological constructs using latent theory (i.e., that psychological symptoms arise from an underlying latent disorder), network theory offers an alternative method for evaluating the relationships between items within a measure [37,38]. Using a network analytic approach, individual symptoms or items are modeled as mutually reinforcing, undirected relationships that give rise to the observed construct or diagnosis [37,38,39]. In these analyses, relationships between individual items (i.e., nodes) are modeled using weighted partial correlations (i.e., edges), allowing for visualization of the relationships between all items in the network while holding all other relationships constant [37,38]. This approach allows one to assess which items are most central to the construct and which items cluster together into subgroups [37]. Through these analyses, critical items may be identified that are more representative of scores on other items in the measure. In other words, an item may be identified as a crucial aspect of empathy as measured by the IRI that is highly related to most other items within the network [37,38]. Furthermore, network comparisons may provide even more detail by juxtaposing whether these central items are the same or different depending upon participant groups (i.e., schizotypy [40]).

Network analyses on the relationships between the specific items of the IRI have been conducted before [41] in non-schizotypy populations. Specifically, Briganti and colleagues [41] found that items from the empathic concern subscale of the IRI were the most central, suggesting that they may be the most significant indicator of overall empathy. Furthermore, items from the fantasy subscale of the IRI were least connected to the other subscales [41]. These results somewhat mirror previous confirmatory factor analyses of the IRI demonstrating that the inclusion of the fantasy subscale does not improve the factor structure [15]. Importantly, additional analyses to assess for differences in networks between high and low schizotypy groups may be highly informative. Based upon previous research showing that the IRI psychometric structure is different between high and low schizotypy groups [15], work suggesting that inter-relations among IRI subscales differ between high and low schizotypy groups [42], and evidence of differing subscale score patterns between participants with and without schizophrenia (of which schizotypy signifies risk for [24]), the structure of the IRI item-level network is likely to vary depending upon the sample’s level of schizotypy.

To forward our understanding of empathy, its facets, and its measurement, our study aimed to conduct a network comparison of the item-level structure of the IRI between high and low schizotypy groups in a large sample of undergraduate students. We hypothesized that the high schizotypy group would evidence a stronger network than the low schizotypy group, consistent with past work [42] and prominent network analysis theories [37]. Namely, the hysteresis principle of network theory postulates that clinical samples typically exhibit stronger networks, as one symptom may activate and maintain others in the network, resulting in a feedback loop of highly related items [37]. Therefore, it is likely that the high schizotypy group, at greater risk for schizophrenia spectrum disorders, may evidence a stronger overall network than the low schizotypy group, which is considered more psychological healthy. Additionally, as previous research has shown the empathic concern subscale to be most central and the fantasy subscale to be least central in a general sample [41], we hypothesized that these centrality results would be replicated in our low schizotypy sample. The structure of the high schizotypy network was considered exploratory.

## 2. Methods

### 2.1. Participants and Procedure

The participants were recruited from two large universities in the Midwest and the Southeast of the United States of America (*n* = 2157), with the participants completing the survey on their respective SONA system platform. Data collection occurred in the periods of 2014–2016 and 2021–2023. The participants were students taking psychology courses who were compensated for their participation in the study through course credit. The eligibility requirements included being 18 years of age or older, being fluent in English, and being a current student at the university. The final sample of participants (*n* = 897) was included in the analyses if their data were of high quality and they met the criteria for the high schizotypy (*n* = 427) or the low schizotypy groups (*n* = 470). The sample was predominately White (75%), female (79%), and non-Hispanic (94.2%). The participants were on average 20.79 (*SD* = 5.18) years old (age range: 18–57 years old).

All study procedures were completed online. The participants completed the survey on an online data collection website (Qualtrics) after providing informed consent. Throughout the survey, ten attention check items were included to ensure the participants were attentive to the survey [43]. Two datasets were combined in order to have a large enough sample of high and low schizotypy participants in order to conduct the appropriate analyses. Very large sample sizes are necessary to have adequate power for network analyses; this study required even larger samples to ensure adequate samples of high and low schizotypy groups. Therefore, this study conducted secondary analyses on data collected from two previous studies (see references [44,45] for more information). In both studies, the participants completed measures of schizotypy and empathy (as well as other measures not included in this paper, as they were not relevant for the current analyses). As the participants did not complete diagnostic interviews with the researchers, information regarding the prevalence of psychiatric diagnoses within this sample cannot be determined. All materials and procedures were approved by the relevant institutional review boards.

### 2.2. Measures

#### 2.2.1. Schizotypy

Schizotypy was assessed using the Schizotypal Personality Questionnaire-Brief Revised (SPQ-BR [46]). The SPQ-BR includes 32 items to measure schizotypal traits on a 5-point Likert scale. Higher scores indicate higher levels of schizotypal traits. The SPQ-BR produces a total score, as well as subscale scores reflecting cognitive–perceptual (“I often feel that others have it in for me”), interpersonal (e.g., “I am not good at expressing my true feelings by the way I talk and look”), and disorganized (e.g., “I have some eccentric (odd) habits”) schizotypy traits. This measure has been found to be both reliable and valid [46,47]. The internal consistency was excellent in our sample (α = 0.95).

#### 2.2.2. Empathy

Empathy was measured using the Interpersonal Reactivity Index (IRI [1,8]). The IRI is a 28-item measure that assesses four facets of empathy, including empathic concern (e.g., “I often have tender, concerned feelings for people less fortunate than me”), personal distress (e.g., “In emergency situations, I feel apprehensive and ill-at-ease”), perspective-taking (e.g., “I try to look at everyone’s side of a disagreement before I make a decision”), and fantasy (e.g., “When I watch a good movie, I can very easily put myself in the place of a leading character”) [1,8]. Within our sample, the internal consistency was acceptable across the subscales (α = 0.73–0.81).

### 2.3. Data Analysis

This network analysis was conducted using secondary analyses of combined data from two separate online studies, which included the measures for the analyses (for detailed information on the separate studies, see references [44,45]). The participants were excluded from data analyses if they frequently endorsed the attention check items. This resulted in a high-quality sample of 1841 participants. Within our study, the participants were categorized into high schizotypy and low schizotypy groups based on their SPQ-BR subscale scores. The participants were included in the high schizotypy group if they scored in the top 10% of respondents on any of the three SPQ-BR subscales. The participants were included in the low schizotypy group only if they scored in the bottom 50% of respondents on all three subscales. These procedures for determining high and low schizotypy groups based on percentiles are consistent with past research in undergraduate samples [15,44,46,48] and align with the level of schizotypy that is thought to confer risk for the development of schizophrenia spectrum disorders [35,49,50]. The descriptive statistics were generated using SPSS version 25. The measures of empathy and schizotypy were assessed for skewness and kurtosis, with values between −2 and +2 being considered acceptable for skewness and from −7 to +7 being considered acceptable for kurtosis according to established guidelines [51]. All measures were found to be normally distributed (see Table S1).

#### 2.3.1. Network Analysis

Networks were estimated for the high and low schizotypy groups separately, and both networks included all items in the IRI. Gaussian graphical models (GGMs) include nodes (i.e., variables of interest; IRI items) and the edges connecting them [52]. These edges represent the partial correlations between pairs of nodes in the model while accounting for the relationships between all nodes in the network simultaneously. In this paper, nodes appear as circles with the amount of exterior shading representing the variance explained by each of the surrounding nodes in the network. The edges appear as lines connecting the nodes, and thicker, darker lines represent stronger correlations. We used the graphical least absolute shrinkage and selection operator (LASSO) to select the optimal tuning parameters in network estimation, shrinking small correlations to zero by minimizing the extended Bayesian information criterion (EBIC [53,54]). Networks were modeled using the *qgraph* package through the R language and environment for statistical computing (v. 4.2.3 [55,56]).

#### 2.3.2. Network Centrality

The overall influence of nodes on the network were estimated using four measures of network centrality. First, measures of closeness assess the length of the shortest paths between nodes. Second, betweenness refers to the sum of shortest paths traveling through the node. Third, strength measures the total weight of a node on the network. Fourth, expected influence calculates the total weight while also accounting for negative correlations [57,58]. Standardized z-scores are calculated for centrality measures and used in figures to assist in the interpretation of the results.

#### 2.3.3. Network Stability and Accuracy

To determine network stability and accuracy, the R package *bootnet* was used. Networks were bootstrapped (1000 iterations), generating confidence intervals for each node in the network. Large confidence intervals indicate increased variability for the nodes in the network and, generally, larger confidence intervals indicate decreased network stability overall. Additionally, networks were subsetted, resampled, and compared to the original sample to calculate the correlation of the subsetted networks to the original samples. Correlation stability (CS) coefficients were calculated for each centrality measure and estimate the proportion of cases that can be dropped and still maintain a 0.7 correlation to the initial model [54]. CS coefficients of 0.25 are generally considered acceptable for interpretation, though coefficients of 0.5 or higher are preferred [54].

#### 2.3.4. Network Comparison

The R package *NetworkComparisonTest* was used to compare high and low schizotypy networks according to three metrics [40]. Network models and stability estimates were developed for both groups separately and network comparison testing was used to assess the network structure invariance, global strength invariance, and edge strength invariance between the two groups using permutation testing (1000 iterations).

#### 2.3.5. Network Community Detection

Network communities were detected using the spinglass algorithm from the *igraph* package in R [59]. The spinglass procedure identifies related nodes in the network (i.e., communities) by using an optimization algorithm that simulates “spins” in a network beginning with an initial random configuration of communities and updating it over many iterations to minimize the energy of the system [60,61]. This leads to a final configuration that represents the optimal community configuration. In this paper, optimal network configurations were indicated by colors.

## 3. Results

### 3.1. Descriptive Statistics

Descriptive statistics for the measures, including skewness and kurtosis, by group are provided in the Supplementary Materials (Table S1). In order to assess whether the time of data collection impacted the empathy scores, independent sample t-tests were run between the two sample sources. The results show that there are very small differences in the subscale scores between the two data collection periods and there is no significant difference between the two samples on any of the four measures of empathy (*p* ≥ 0.05). The descriptive statistics of the IRI by data collection period as well as the independent sample t-test results are available in the Supplementary Materials (Tables S2 and S3). As expected, high schizotypy participants (*M* = 119.34) had significantly higher schizotypy scores than low schizotypy participants (*M* = 67.44), *t*(678) = −47.55, *p* < 0.001. High schizotypy participants scored significantly higher on the IRI fantasy subscale (*M* = 27.02) than the low schizotypy participants (*M* = 22.96), *t*(667) = −8.51, *p* < 0.001. High schizotypy participants also scored significantly higher on the IRI distress subscale (*M* = 22.00) than the low schizotypy group (*M* = 18.38), *t*(663) = −9.76, *p* < 0.001. High and low schizotypy participants did not significantly differ in their scores on the IRI perspective-taking (*M* = 25.25 and 25.09, respectively; *p* = 0.69) or the empathic concern (*M* = 27.36 and 27.22, respectively; *p* = 0.70) subscales. The results of network analysis assessing the structure of the IRI with the full high-quality sample can be seen in Supplementary Figures (Figures S1–S3).

### 3.2. Network of Interpersonal Reactivity Index Items in the Low Schizotypy Group

**Figure 1:**
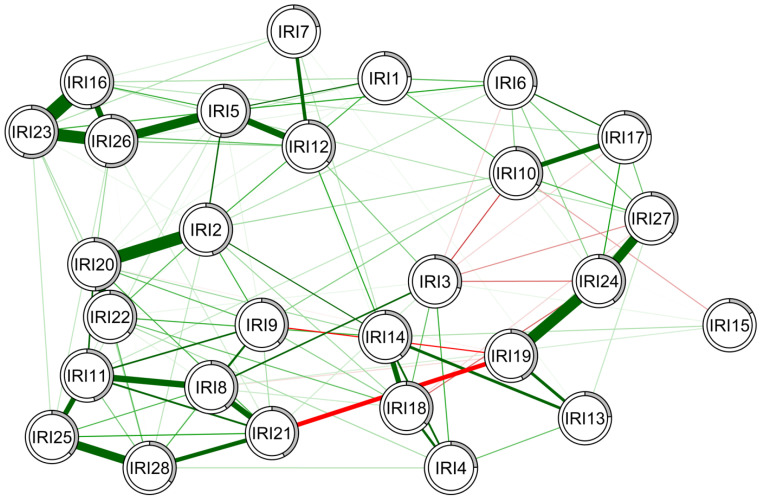
Estimated network structure of IRI items in the low schizotypy group. Edges in green indicate positive partial correlations, while the red lines indicate negative ones. The thicker the line, the stronger the connection. The white ring around the nodes shows the amount of variance that the item covers. IRI: Interpersonal Reactivity Index.

For the low schizotypy group, a network structure was developed for IRI item responses (Figure 1). Lambda parameters for this sample were 0.11. The edge stability was assessed (Figures S4 and S5) by estimating the variability in the edge strength in 1000 bootstrap samples, generating 95% confidence intervals. These figures included moderate confidence interval widths, suggesting relatively stable edge strengths in the samples. After bootstrapping, the CS coefficients for expected influence (CS = 0.52) were greater than 0.5 and the CS coefficients for betweenness (CS = 0.28) and strength (CS = 0.44) were greater than 0.25, indicating relative stability and that they can be reasonably assessed. Based on these findings, IRI items 2 (b = 78, ei = 0.95, s = 0.91), 5 (b = 102, ei = 1.02, s = 1.02), 20 (b = 62, ei = 0.99, s = 1.00), 23 (b = 0, ei = 0.95, s = 0.95), and 26 (b = 46, ei = 0.91, s = 0.91) had the greatest influence on the overall model. Additionally, particularly strong edge correlations were found between IRI items 16 and 23, 23 and 26, 2 and 20, and 19 and 24. Overall, IRI 5 had the greatest influence on the model, as indicated by betweenness (b = 102), expected influence (ei = 1.02), and strength (s = 1.02) (Figure S6). IRI 15 had the weakest influence on the model, as indicated by expected influence (ei = 0.11).

### 3.3. Network of Interpersonal Reactivity Index Items in the High Schizotypy Group

**Figure 2:**
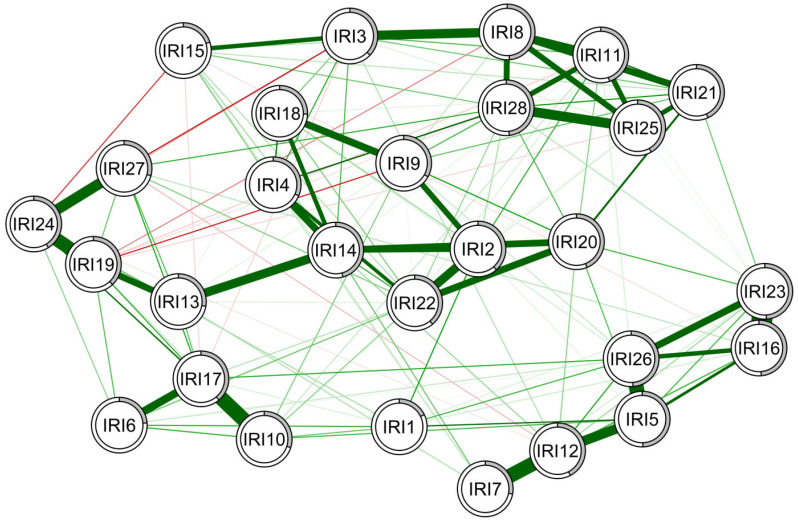
Estimated network structure of IRI items in the high schizotypy group. Edges in green indicate positive partial correlations, while the red lines indicate negative ones. The thicker the line, the stronger the connection. The white ring around the nodes shows the amount of variance that the item covers. IRI: Interpersonal Reactivity Index.

A network structure was developed for IRI item responses within only the high schizotypy group as well (Figure 2). The edge stability assessed using 1000 bootstrap samples, generating 95% confidence intervals (Figures S7 and S8). The lambda parameters for this sample were 0.10. The confidence intervals were moderate, suggesting relative edge stability. After bootstrapping, the CS coefficients were greater than 0.5 for expected influence (CS = 0.52) and strength (CS = 0.52), indicating that they are stable and can be reasonably assessed. IRI items 5 (ei = 1.06, s = 1.06), 14 (ei = 1.22, s = 1.22), 20 (ei = 0.98, s = 0.98), 26 (ei = 0.98, s = 0.98), and 28 (ei = 1.09, s = 1.12) had the greatest influence on the model. Additionally, strong edges existed between IRI items 10 and 17, 7 and 12, and 19 and 24. When examining subset bootstraps, IRI 14 had the greatest influence on the model, assessed by expected influence (ei = 1.22) and strength (s = 1.22) (Figure S9). IRI 15 had the weakest influence on the model, as determined by expected influence (ei = 0.3).

### 3.4. Network Comparison of the Interpersonal Reactivity Index Items between the High and Low Schizotypy Groups

**Figure 3:**
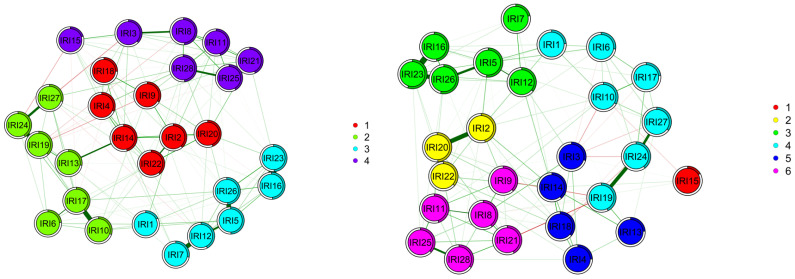
Estimated network comparisons of IRI items in the high (**left**) and low schizotypy (**right**) groups. Edges in green indicate positive partial correlations, while the red lines indicate negative ones. The thicker the line, the stronger the connection. The white ring around the nodes shows the amount of variance that the item covers. The IRI items were forced into best-fit groups for both the high and low schizotypy groups. High schizotypy was settled into 4 groups, while low schizotypy was settled into 6 groups. IRI: Interpersonal Reactivity Index.

In the low schizotypy group, the IRI items clustered into six separate groups (Figure 3): Cluster 1—RI item 15; Cluster 2—IRI items 2, 20, and 22; Cluster 3—IRI items 5, 7, 12, 16, 23, and 26; Cluster 4—IRI items 1, 6, 10, 17, 19, 24, and 27; Cluster 5—IRI items 3, 4, 13, 14, and 18; and Cluster 6—IRI items 8, 9, 11, 21, 25, and 28. In the high schizotypy group, the IRI items were clustered into four groups that were aligned with the IRI subscales. After conducting a centrality difference test (Figure S10), IRI item 17 differed significantly between the two networks (EI *p* = 0.03, *p* < 0.001). This difference was evidenced by a greater strength (s = 1.01), betweenness (b = 47), and expected influence (ei = 0.95) in the high schizotypy network than the low schizotypy network (b = 22, c = 0.002, EI = 0.49, Strength = 0.52). The edges were not significantly different between the networks (*p* > 0.05). Network structure comparisons, using tests for invariance and 1000 bootstrap samples, showed significantly different structures (M = 0.21, *p* = 0.05), but not a significantly different global strength (S = 2.05, *p* = 0.01) between the two networks.

Additionally, recent research has suggested that alternative methods of community detection, specifically using the “walktrap” algorithm, may be useful for interpretation [62]. These analyses were conducted for the high and low schizotypy samples and are included in the Supplementary Materials. The high schizotypy networks in the two community detection models were equivalent. The low schizotypy network using the “walktrap” algorithm evidenced four communities, rather than six, with three communities from the spinglass model being grouped together. 

## 4. Discussion

This study sought to assess the item-level network structure of the Interpersonal Reactivity Index (IRI [1,8]) in participants with high and low schizotypy. Inconsistent with hypotheses, the results show that the overall strengths of the networks are statistically similar. However, the network structures between the groups differed. Further analysis of the community structure of nodes within the networks indicated that the high schizotypy group mirrored that of the IRI subscales, with items from each subscale clustered together and distinct from the other subscale clusters. By contrast, the network of the low schizotypy group evidenced six clusters that did not replicate the IRI subscales. In the high schizotypy group, items from the empathic concern subscale were the most central to the network, whereas the low schizotypy group network had a relative lack of centrality.

Appropriate assessment of empathy is important broadly, but particularly in high schizotypy groups. As high schizotypy groups experience social functioning deficits [31,32,33], the identification of those who may be at greater risk for these negative outcomes through empathy assessment could aid in intervention. Through network analyses, influential items that were highly representative of responses across the measure were identified. For the high schizotypy group, of the five items with the greatest influence on the model, two items were from the fantasy subscale, two from the empathic concern subscale, and one from the perspective-taking subscale. Of these, an item from the empathic concern subscale had the greatest expected influence (IRI 14: “Other people’s misfortunes do not usually disturb me a great deal”). The replication of the IRI subscales in the network analysis suggests that the IRI items function as would be expected from the original scale for the high schizotypy group, but not the low. This highlights that additional work is needed to clarify these findings and recent confirmatory factor analyses showing that psychometric properties were inadequate when including all four subscales [15].

The low schizotypy IRI network showed a clustering that did not correspond to the traditional four-subscale structure of the IRI. Within the low schizotypy network, six clusters emerged. One cluster included only one reverse-scored item from the perspective-taking subscale (IRI 15: “If I’m sure I’m right about something, I don’t waste much time listening to other people’s arguments”). The remaining five clusters were composed of items from a variety of subscales. For instance, cluster five included items from three of the subscales. There were no clusters that included items from only one of the four distinct subscales originally included in the IRI. Therefore, the results suggest that the participants’ item-level responses were inter-related in unexpected ways that do not map onto any existing subscale structures currently in use. One may be tempted to interpret this as suggesting a total score for the IRI would be most appropriate; however, a vast body of literature has established that a total score is inappropriate for the IRI and would render scores uninterpretable [1,8,15,63]. Thus, future work should investigate the utility of other subscale structures and whether item 15 should be included in the measure for those with low schizotypy.

Interestingly, three items demonstrated the greatest influence within both the low and high schizotypy networks. These included two items from the fantasy subscale (IRI items 5 (“I really get involved with the feelings of the characters in a novel”) and 26 (“When I am reading an interesting story or novel, I imagine how I would feel if the events in the story were happening to me”)) and one item from the empathic concern subscale (IRI item 20 (“I am often quite touched by things that I see happen”)). While previous work has suggested that the fantasy subscale should not be included when administering the IRI to those with high schizotypy [15], the results from this network analysis highlight that, when the full scale is administered, these two fantasy items may be particularly good indicators of how participants will respond to other items across both the high and low schizotypy groups.

Despite this, debate exists on whether the fantasy subscale is measuring empathy or a separate, but related, construct that may be more closely linked to psychosis than other facets of empathy [64,65,66]. However, our results could indicate that, on the IRI, one’s ability to imagine themselves in the place of a fictional character has high influence on how one responds to other IRI items, including those more closely aligned with core empathy-related concepts. Additional work could clarify and extend this finding by examining if they extend to other, at risk for psychosis groups, such as those at clinical high risk for psychosis.

Furthermore, one item from the perspective-taking subscale demonstrated the *lowest* expected influence in both the high and low schizotypy networks (IRI item 15 (“If I’m sure I’m right about something, I don’t waste much time listening to other people’s arguments”)). This suggests that this item may be a particularly poor indicator of how participants may respond to other items in this measure. One explanation for the low influence of this item may be that this item includes a stronger negative connotation towards others than other items in the IRI. Therefore, the wording of this item may lead participants to feel hesitant to agree with this statement, with only those who have extremely low empathic tendencies endorsing it. Additional research should assess whether this item accurately reflects an aspect of one’s level of perspective-taking empathy.

Regarding centrality, one item from the personal distress scale (IRI item 17 (“Being in a tense emotional situation scares me”)) was significantly more central to the high schizotypy network than the low schizotypy network. This suggests that this item is more predictive of response patterns in the high schizotypy group than the low schizotypy group. For high schizotypy participants, this item may be more related to empathic abilities measured through the other items. Unsurprisingly, the items most strongly related to IRI item 17 (IRI items 6 and 10) are also from the personal distress subscale. Previous research has illustrated that schizophrenia spectrum participants evidence higher personal distress scores than the healthy controls, perhaps due to difficulties in emotion regulation and distress tolerance, and lower levels of empathy [24,67]. Further evaluation of whether this pattern of increased personal distress extends to those high in schizotypy is warranted.

Against our hypothesis, the high schizotypy group did not demonstrate a stronger network structure than the low schizotypy group. While our hypothesis was based on previous findings [33] and the hysteresis principle of network theory [37], works suggesting stronger network structures in groups with psychopathology have largely examined networks with larger constructs as nodes [68,69]. This same phenomenon of stronger networks may not hold for item-level networks of one very specific aspect of social functioning (i.e., empathy), where the hysteresis principle of network theory stating that symptoms exacerbate one another may be less applicable. Furthermore, though high schizotypy has been associated with other negative mental health outcomes [70,71,72], including in undergraduate populations [73,74], it is possible that the degree of difference in psychological health between the high and low schizotypy groups in this sample was not great enough to be reflected in the network structures as would have been predicted by the hysteresis principle [37].

Our study has limitations. First, our study included only undergraduate students, which may limit the generalizability of our results. However, this is consistent with considerable research on high schizotypy (e.g., [46,48,75,76]). Second, our study was limited by only including one measure of empathy. While the IRI is one of the most widely-used measures of empathy [9], our work informs only studies using the IRI and not the broader body of empathy research. There are many other measures of empathy [76,77], including those built from alternate empathy conceptualizations [78,79,80,81,82], which our results cannot inform. Third, our study included participants in the high schizotypy group if they scored in the top 10% on any of the three schizotypy subscales. Some schizotypy research using undergraduate samples elected to include the top 5% of respondents in order to account for college samples having a higher average functioning. We elected to include the top 10% to increase the statistical power and the generalizability of our results. Fourth, due to the online nature of this study with a large sample, diagnostic interviews were not conducted with the participants, which precludes any conclusions regarding the network structure of the IRI in a sample of peoples with diagnosed Schizotypal Personality Disorder. Future research should be conducted to assess the network structures of empathy in this group. Fifth, network analyses present many options for comparison of networks that may influence the results somewhat. Our high and low GLASSO networks evidenced slightly different but ultimately comparable lambda parameters (0.10 and 0.11, respectively). While GLASSO networks are widely considered to be appropriate for these comparisons, future studies should assess IRI networks using alternative methods, such as informational filtering networks. Sixth, the long period of data collection (2014–2023) may have allowed for changes in lifestyles to impact our results. While comparisons between the early collected data and the more recently collected data did not suggest significant differences in empathy, future network analyses assessing empathy may benefit from large datasets collected in a short timeframe to avoid this limitation.

The implications of this research suggest the use of the IRI to determine levels of empathy when related to schizotypy may be inappropriate, as research is conflicting in whether the four-factor structure is appropriate for use with high schizotypy groups [15] and network-found clusters of items that do not mirror the four-factor structure in the low schizotypy group. Together, these findings indicate that the current IRI structure may not perform adequately with either the low or high schizotypy group.

While this study provides important insights into the use of the IRI in high and low schizotypy groups, many areas of future research exist. For example, work should be conducted to assess the item-level structure of the IRI in a sample of participants with clinical diagnoses relevant to empathy impairments, including schizophrenia spectrum and autism spectrum disorders and antisocial personality disorder. Additional work should be conducted to assess variance in the network structure between high and low schizotypy groups in other measures of empathy. Furthermore, work should be conducted to assess the network structure of the IRI in groups high and low in other personality traits relevant to empathy, such as antisocial traits.

## 5. Conclusions

This study assessed the item-level network of the IRI and whether network structures varied between high and low schizotypy groups. Our results suggest that the item-level structure of the IRI may be dependent upon the level of schizotypy of the sample in which it is being used, and further research is needed to determine the factor structure of the IRI in these groups. Both the high and low schizotypy groups had the same three items that were the most predictive and one item that was the least predictive of scores on the other items. In contrast to these similarities, the low schizotypy group did not evidence an item structure that aligned with any known factor structure of the measure, but rather produced six clusters that were not in line with the established factor structures, suggesting that further analyses of the IRI are needed. By contrast, the high schizotypy group evidenced four clusters that aligned with the established four-factor structure. These results highlight that our understanding of how different facets of empathy are related to each other may be dependent upon personality factors such as schizotypy, which are at times overlooked.

## Data Availability

The data presented in this study are available upon request from the corresponding author. The data are not publicly available.

## Supplementary Materials

The following supporting information can be downloaded at: https://www.mdpi.com/article/10.3390/bs14030245/s1, Figure S1: Estimated network structure of IRI items in the full high-quality sample; Figure S2: Bootstrapped confidence intervals and edge weights for the full high-quality sample network; Figure S3: Subsetting bootstrap for the full high-quality sample network demonstrating average centrality estimates for the original networks relative to the subsetted estimates with fewer samples; Figure S4: Bootstrapped confidence intervals and edge weights for the low schizotypy network; Figure S5: Subsetting bootstrap for the low schizotypy network demonstrating average centrality estimates for the original networks relative to the subsetted estimates with fewer samples; Figure S6: Centrality indexes of each node in the low schizotypy group estimated network structure; Figure S7: Bootstrapped confidence intervals and edge weights for the high schizotypy network; Figure S8: Subsetting bootstrap for the high schizotypy network demonstrating average centrality estimates for the original networks relative to the subsetted estimates with fewer samples; Figure S9: Centrality indexes of each node in the high schizotypy group estimated network structure; Figure S10: Centrality indexes of each node in the estimated network comparison structure; Figure S11: Community detection of IRI items in the low schizotypy group assessed using the “walktrap” algorithm; Figure S12: Community detection of IRI items in the high schizotypy group assessed using the “walktrap” algorithm; Table S1: Descriptive statistics of measures by group; Table S2: Descriptive statistics of the interpersonal reactivity index by data collection period; Table S3: Results of independent samples t-tests of IRI by data collection period.
