# Supplementary material for: Empathy and Schizotypy: A Network Comparison of the Interpersonal Reactivity Index in High and Low Schizotypy Groups
Source: Behav Sci (Basel). 2024 Mar 18;14(3):245. doi: 10.3390/bs14030245 (PMC10968485; doi:10.3390/bs14030245)
Supplement: Supplementary file 1 [file behavsci-14-00245-s001.zip › behavsci-2872600-supplementary.pdf]

Table S1. Descriptive statistics of the measures by group.

		IRI-F	IRI-PT	IRI-EC	IRI-D	SPQ-T	SPQ-I	SPQ-D	SPQ-CP
Total Sample (*n* = 1841)	*M* (*SD*)	24.27 (5.68)	25.06 (4.52)	27.02 (4.52)	19.76 (4.56)	90.39 (19.98)	29.46 (7.76)	24.88 (6.45)	35.95 (9.88)
	Min–Max	8-35	9-35	8-35	7-34	32-155	6-50	6-40	14-66
	Skewness	-0.15	-0.11	-0.35	-0.20	-0.12	-0.12	-0.18	0.08
	Kurtosis	-0.45	-0.10	-0.12	-0.09	0.01	-0.24	-0.25	-0.28
Low Schizotypy (*n* = 470)	*M* (*SD*)	22.96 (5.65)	25.09 (4.33)	27.22 (4.39)	18.38 (4.30)	67.44 (12.54)	22.49 (5.41)	18.52 (4.58)	26.43 (6.16)
	Min–Max	8-35	13-35	11-35	7-32	32-91	6-30	6-25	14-36
	Skewness	-0.03	0.01	-0.35	-0.15	-0.63	-0.58	-0.53	-0.30
	Kurtosis	-0.41	-0.28	0.01	-0.05	-0.09	-0.46	-0.54	-0.91
High Schizotypy (*n* = 427)	*M* (*SD*)	26.28 (5.79)	25.13 (5.13)	27.58 (4.80)	21.32 (4.81)	113.06 (12.68)	36.36 (7.30)	31.14 (5.28)	45.56 (8.72)
	Min–Max	8-35	9-35	8-35	7-34	82-155	9-50	11-40	20-66
	Skewness	-0.58	-0.34	-0.56	-0.44	-0.00	-0.84	-0.80	-0.42
	Kurtosis	0.04	-0.13	-0.01	0.10	0.04	0.54	0.64	0.08

Note. IRI-F = Interpersonal Reactivity Index—Fantasy subscale; IRI-PT = Interpersonal Reactivity Index—Perspective-Taking subscale; IRI-EC = Interpersonal Reactivity Index—Empathic Concern subscale; IRI-D = Interpersonal Reactivity Index—Distress subscale; SPQ-T = Schizotypal Personality Questionnaire-Brief Revised-Total score; SPQ-I = Schizotypal Personality Questionnaire-Brief Revised-Interpersonal score; SPQ-D = Schizotypal Personality Questionnaire-Brief Revised-Disorganization score; SPQ-CP = Schizotypal Personality Questionnaire-Brief Revised-Cognitive–Perceptual score.

Table S2. Descriptive statistics of the Interpersonal Reactivity Index by data collection period.

**Subscale**	**Data Collection**	***N***	***M***	***SD***
IRI- F	2021-2023	892	23.39	5.81
	2014-2016	917	24.14	5.56
IRI- PT	2021-2023	883	25.25	4.58
	2014-2016	909	24.88	4.46
IRI- EC	2021-2023	888	27.23	4.52
	2014-2016	899	26.81	4.52
IRI- D	2021-2023	887	19.55	4.78
	2014-2016	902	19.97	4.33

Note. IRI-F = Interpersonal Reactivity Index—Fantasy subscale; IRI-PT = Interpersonal Reactivity Index—Perspective-Taking subscale; IRI-EC = Interpersonal Reactivity Index—Empathic Concern subscale; IRI-D = Interpersonal Reactivity Index—Distress subscale.

Table S3. Results of the independent sample t-tests of the IRI by data collection period.

**Subscale**	***t***	***df***	***p***
IRI-F	0.92	1807	0.36
IRI-PT	1.72	1790	0.09
IRI-EC	1.98	1785	0.05
IRI-EC	-1.97	1787	0.05

Note. IRI-F = Interpersonal Reactivity Index—Fantasy subscale; IRI-PT = Interpersonal Reactivity Index—Perspective-Taking subscale; IRI-EC = Interpersonal Reactivity Index—Empathic Concern subscale; IRI-D = Interpersonal Reactivity Index—Distress subscale.

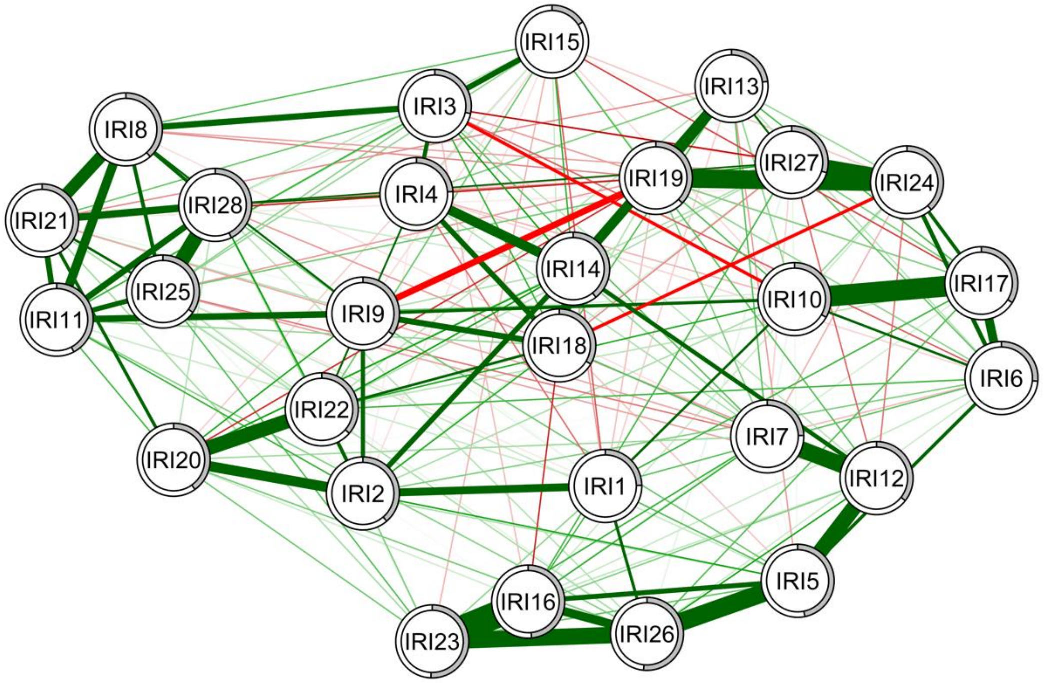

Figure S1. Estimated network structure of IRI items in the full high-quality sample. Edges in green indicate positive partial correlations, while red lines indicate negative ones. The thicker the line, the stronger the connection. The white ring around the nodes shows the amount of variance that item covers. IRI: Interpersonal Reactivity Index.

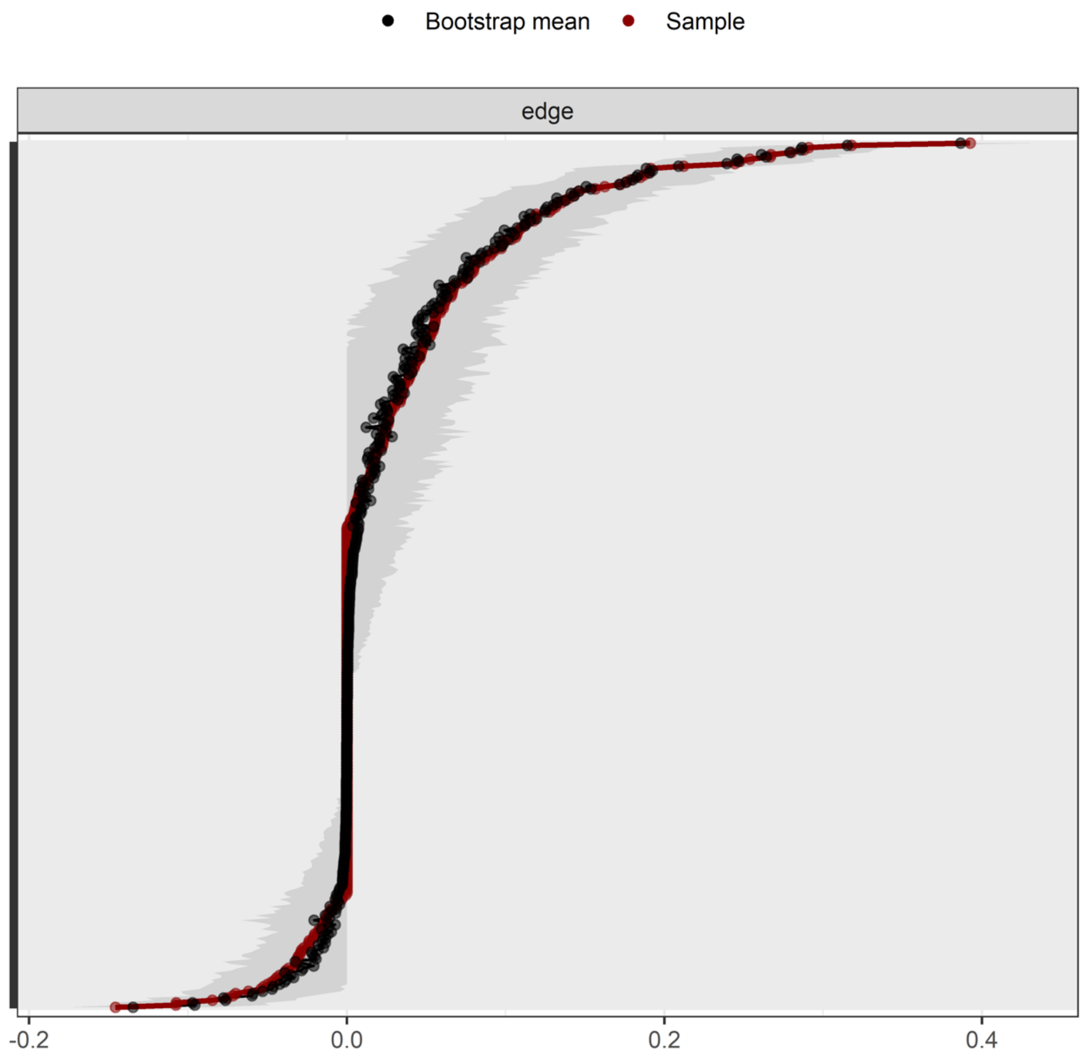

Figure S2. Bootstrapped confidence intervals and edge weights for the full high-quality sample network.

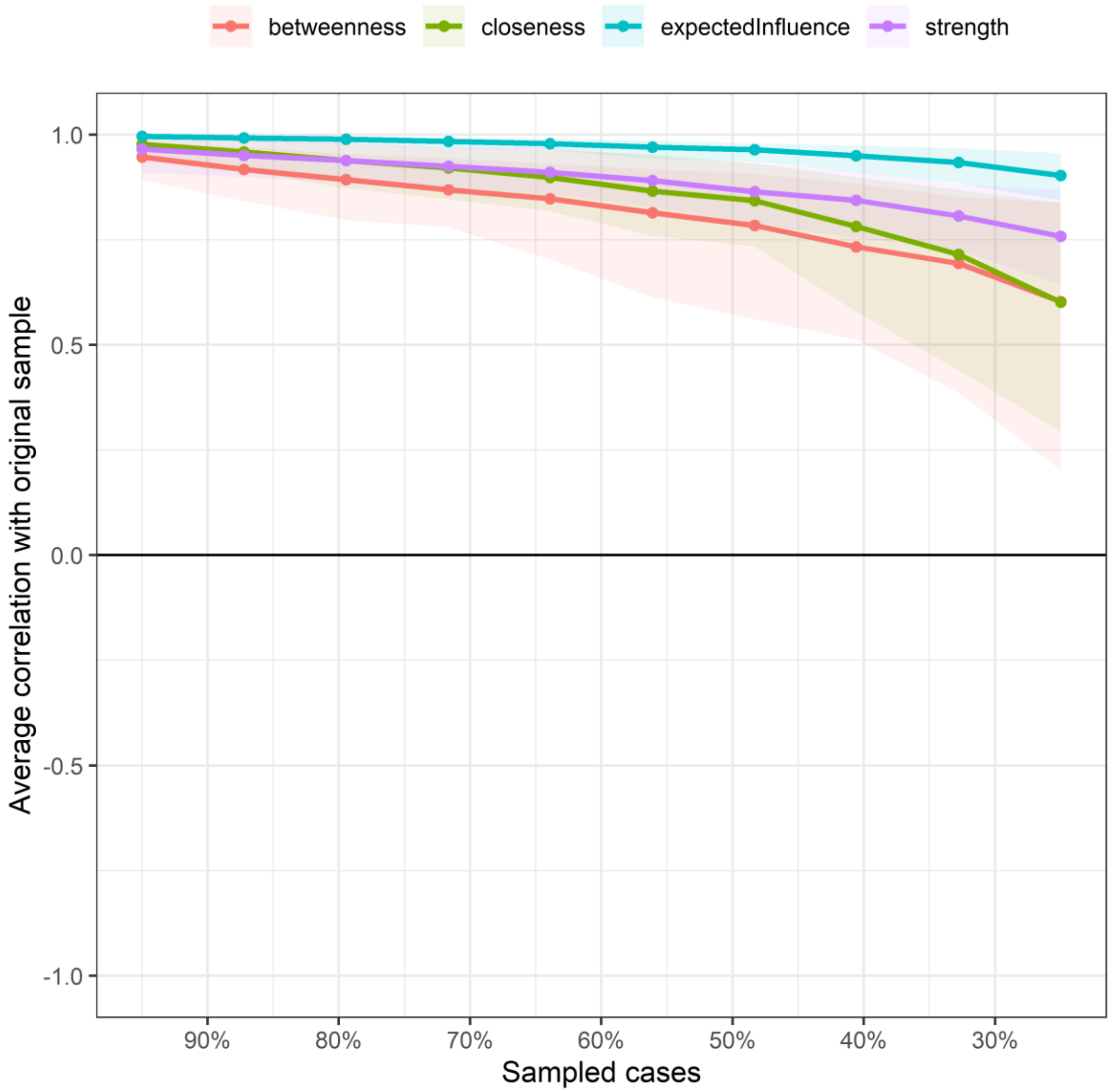

Figure S3. Subsetting bootstrap for the full high-quality sample network demonstrating average centrality estimates for the original networks relative to the subsetted estimates with fewer samples.

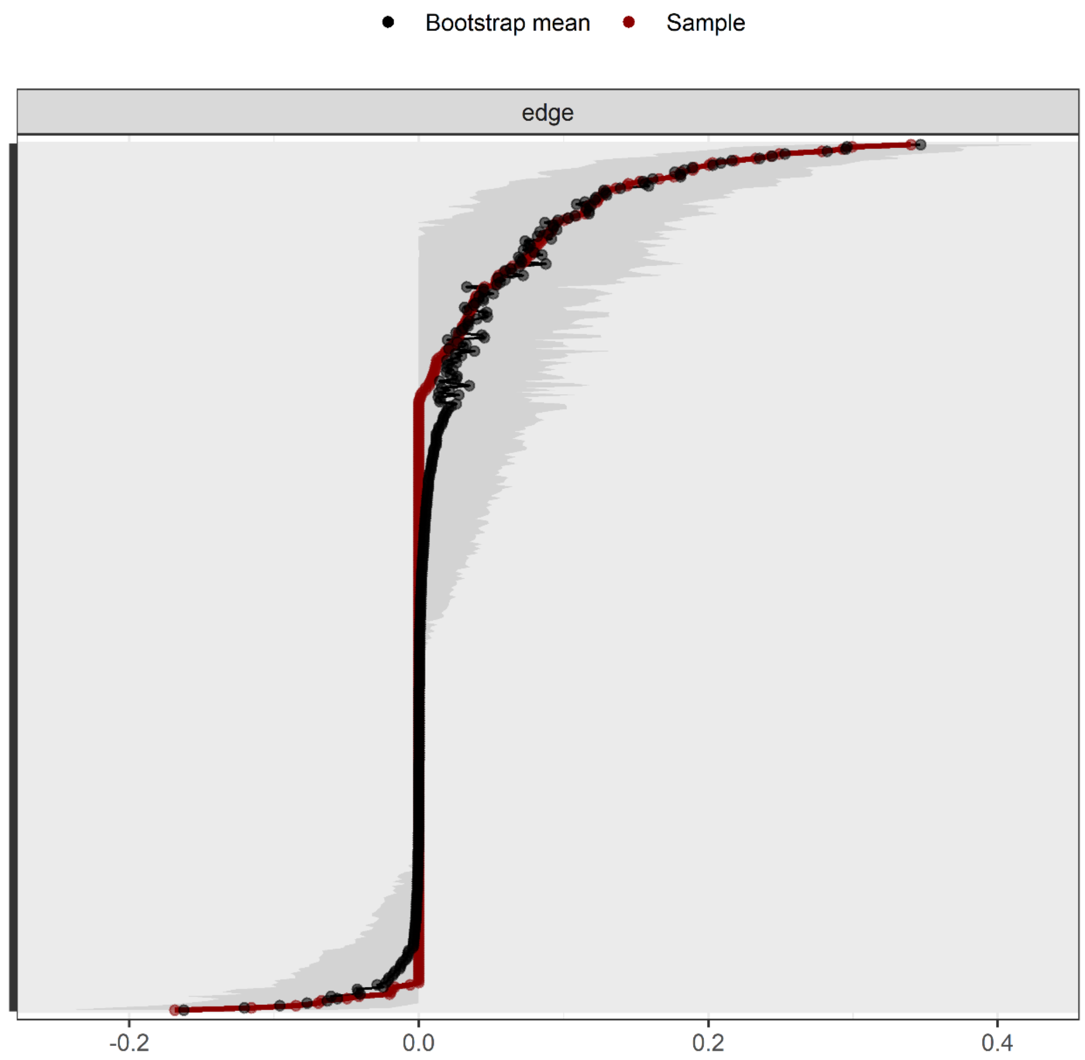

Figure S4. Bootstrapped confidence intervals and edge weights for the low schizotypy network.

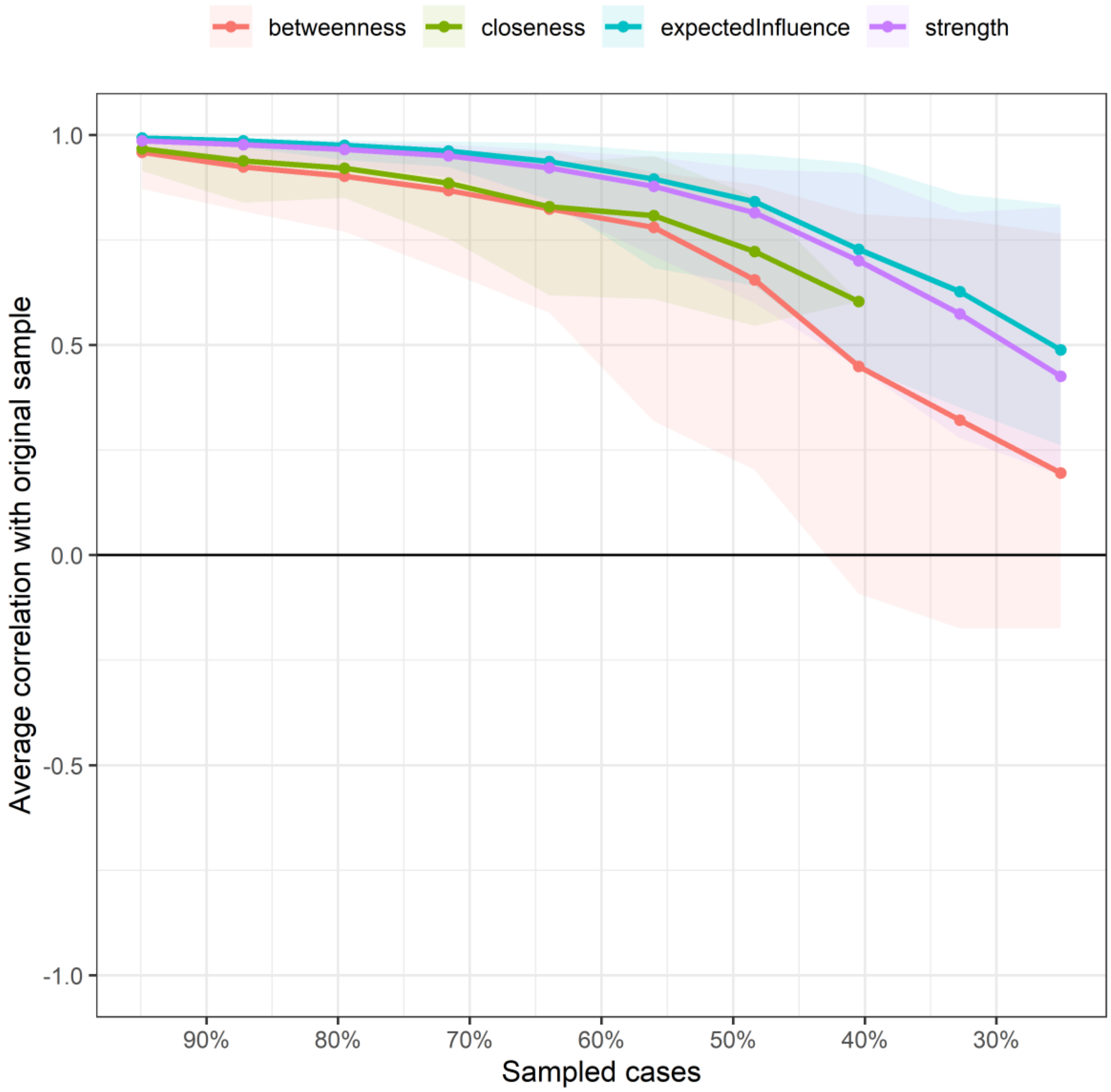

Figure S5. Subsetting bootstrap for the low schizotypy network demonstrating average centrality estimates for the original networks relative to the subsetted estimates with fewer samples.

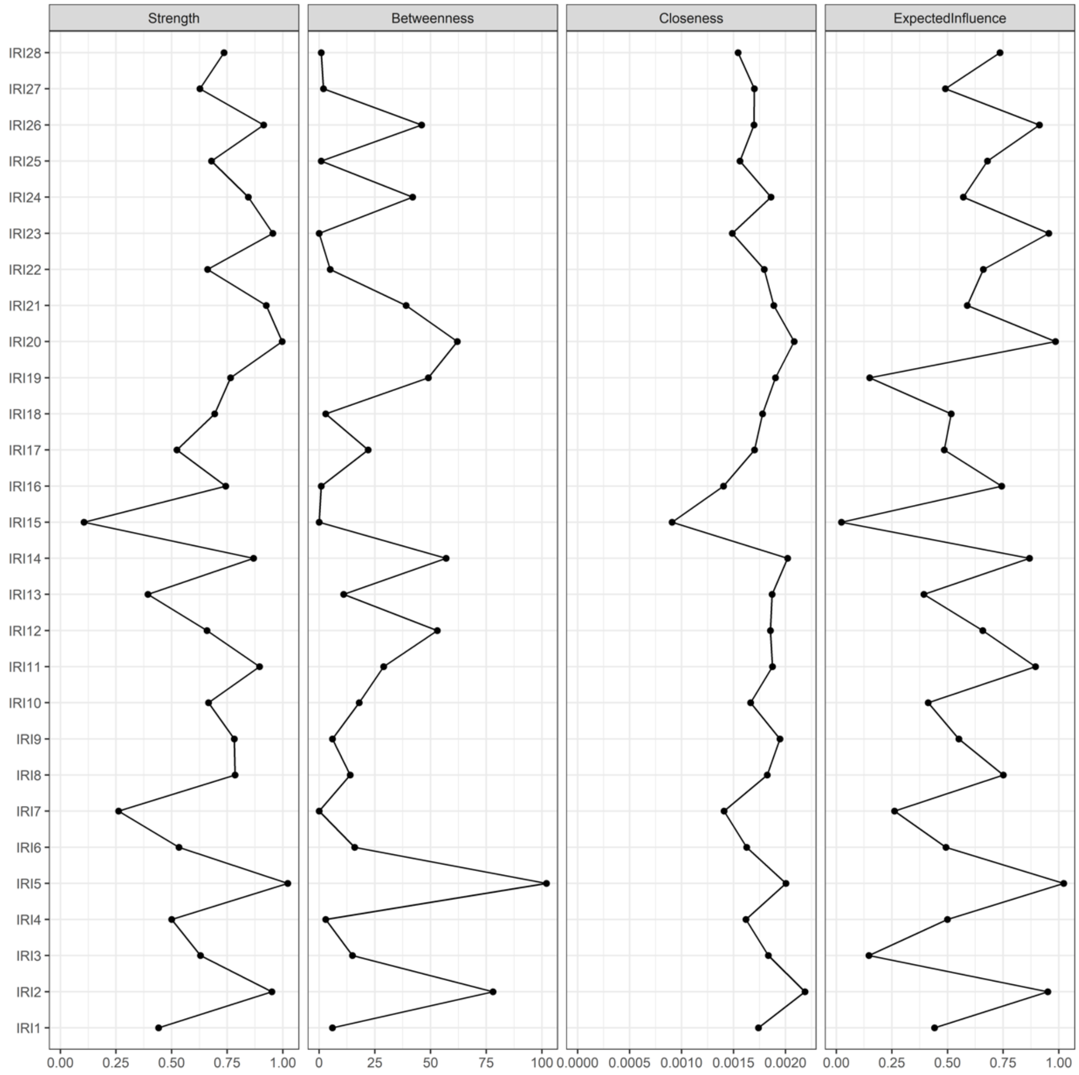

Figure S6. Centrality indexes of each node in the low schizotypy group estimated network structure. Centrality indexes are represented by z-scores.

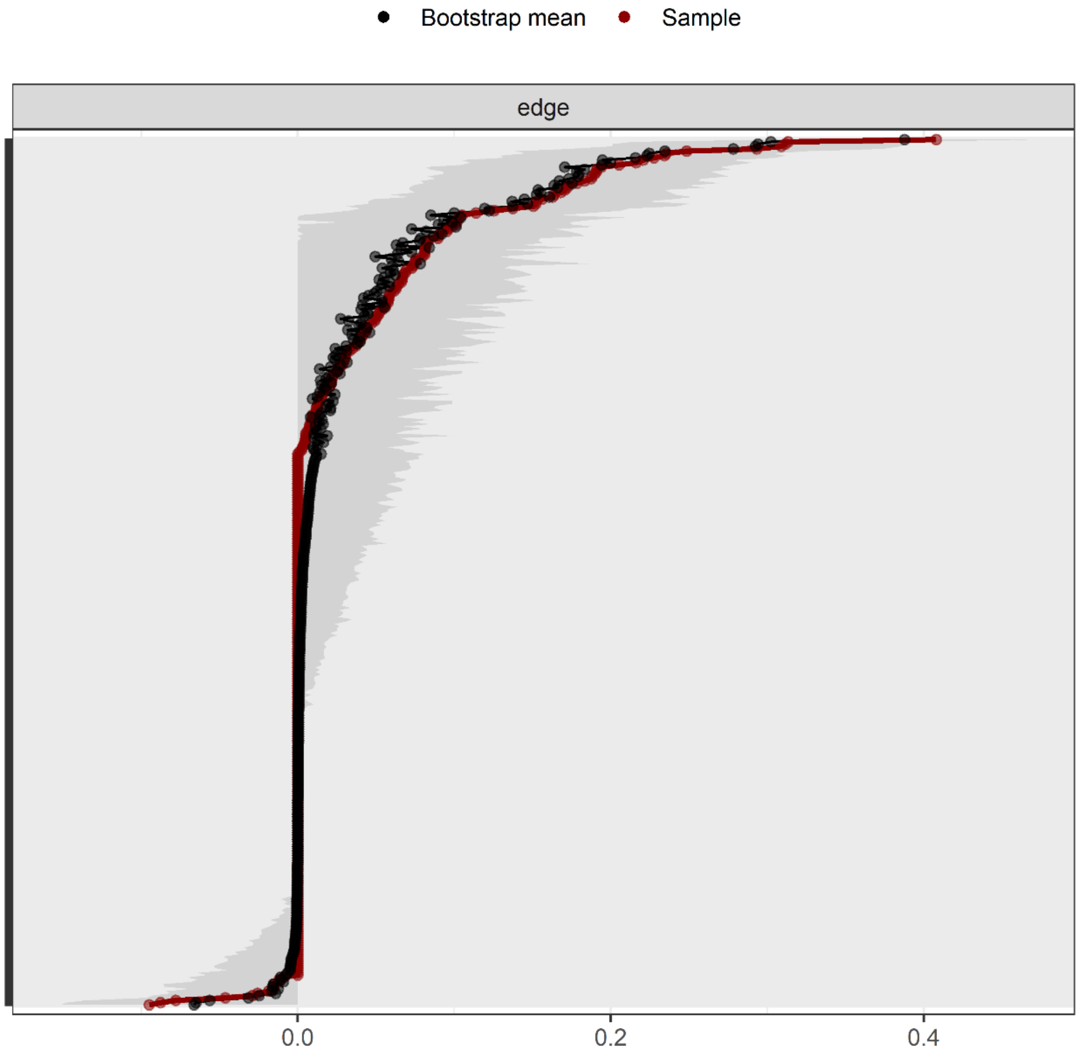

Figure S7. Bootstrapped confidence intervals and edge weights for the high schizotypy network.

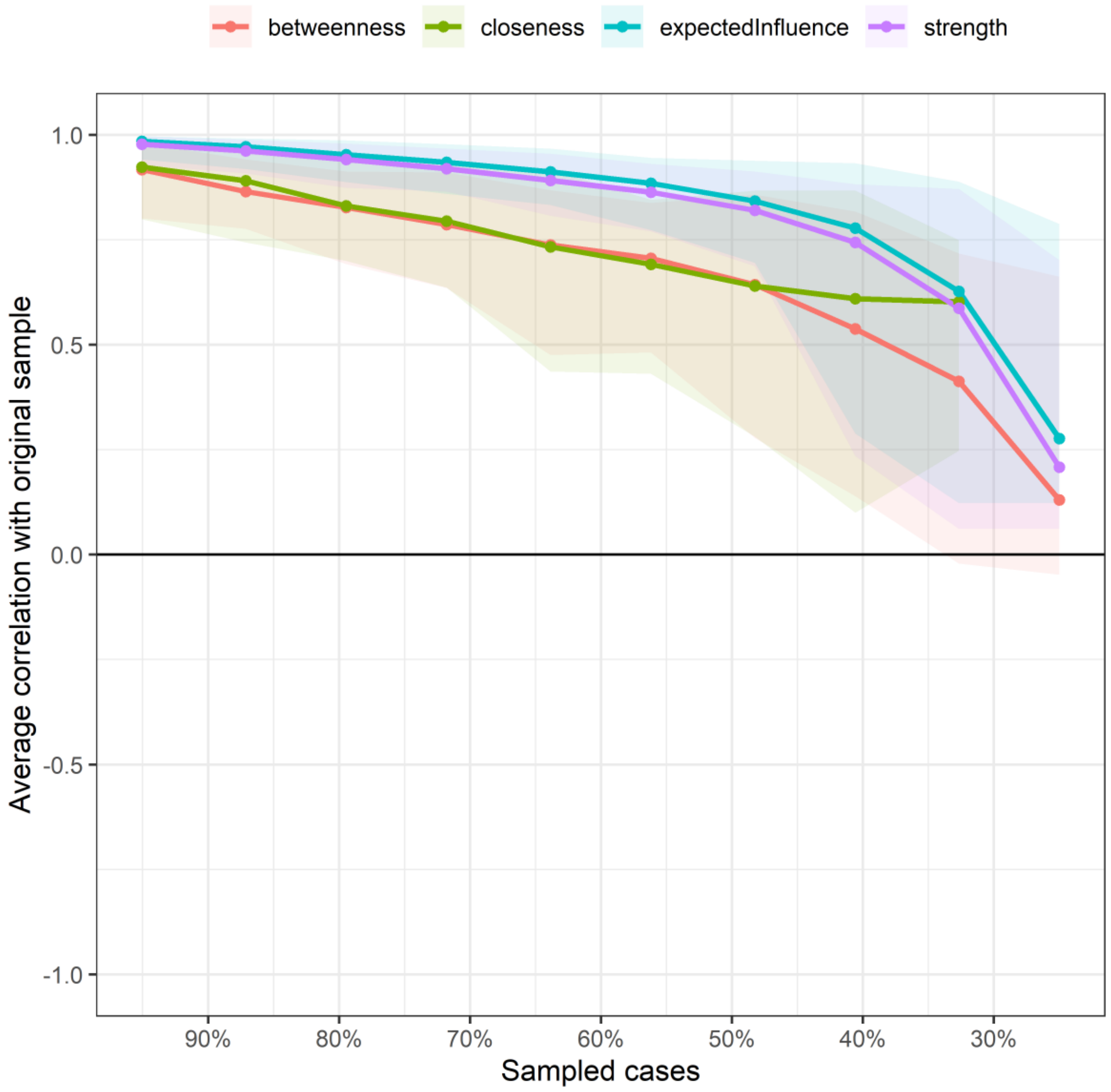

Figure S8. Subsetting bootstrap for the high schizotypy network demonstrating average centrality estimates for the original networks relative to the subsetted estimates with fewer samples.

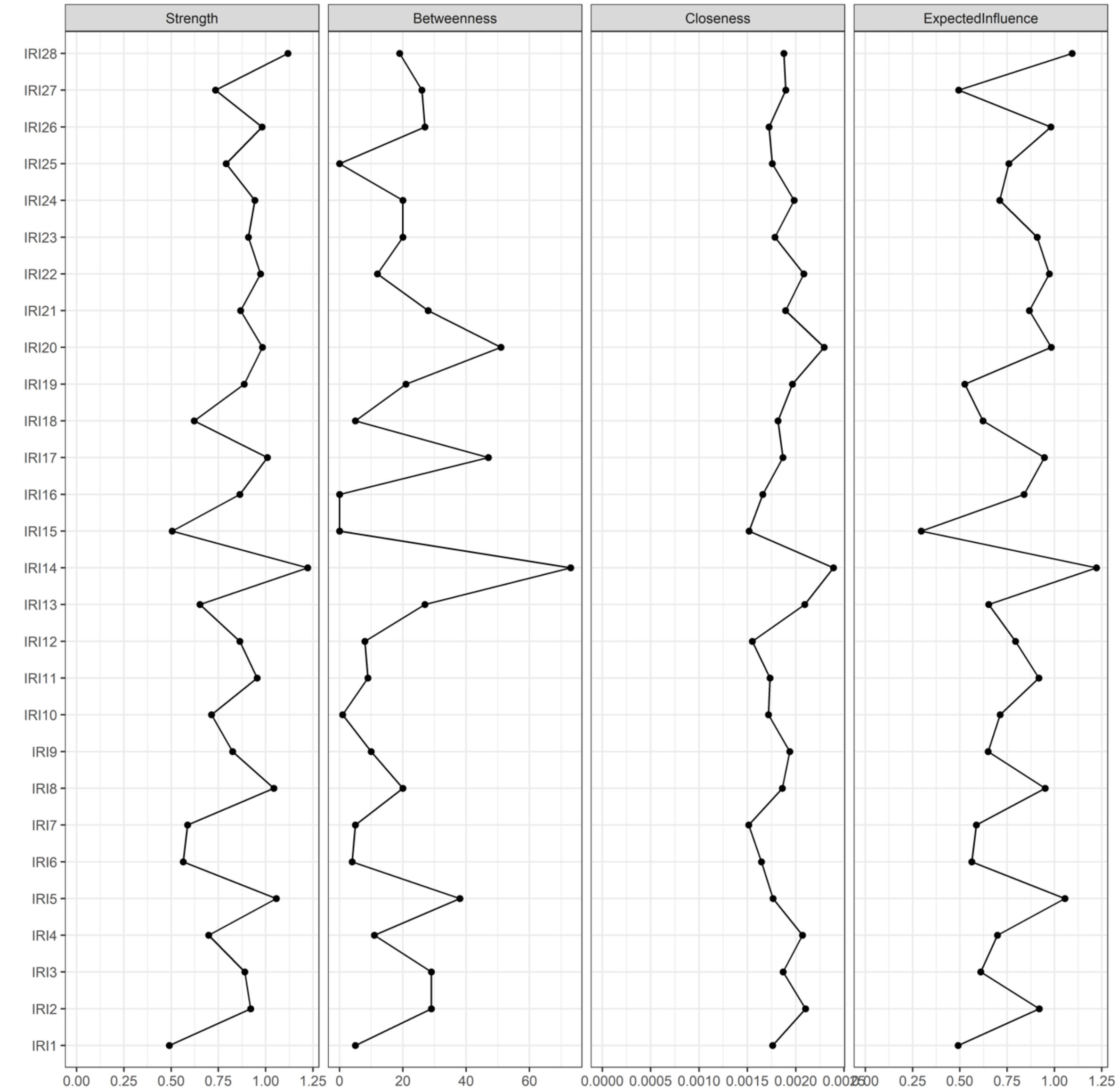

Figure S9. Centrality indexes of each node in the high schizotypy group estimated network structure. Centrality indexes are represented by z-scores.

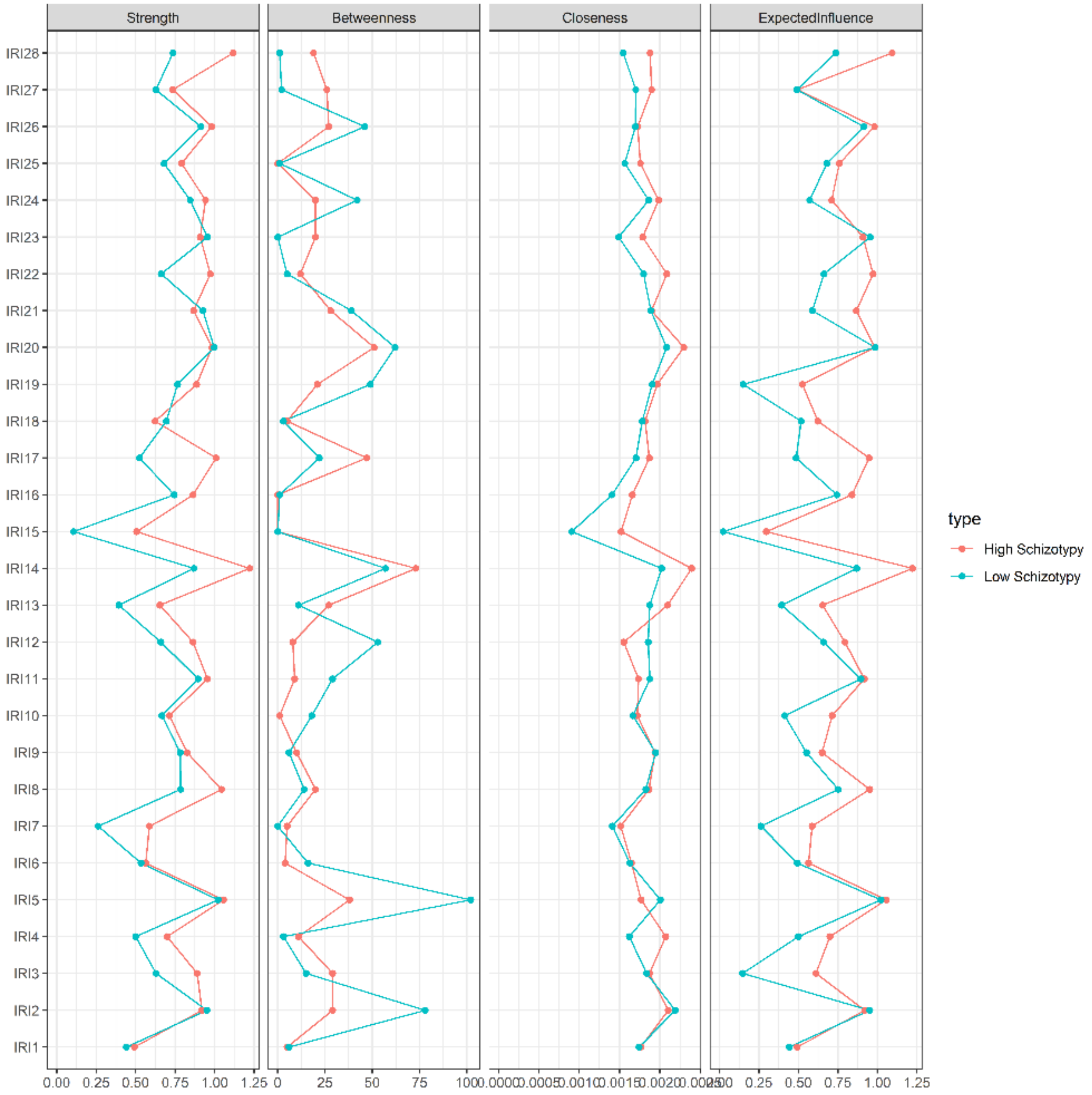

Figure S10. Centrality indexes of each node in the estimated network comparison structure. Centrality indexes are represented by z-scores. Z-scores for each group are overlayed for easy comparison.

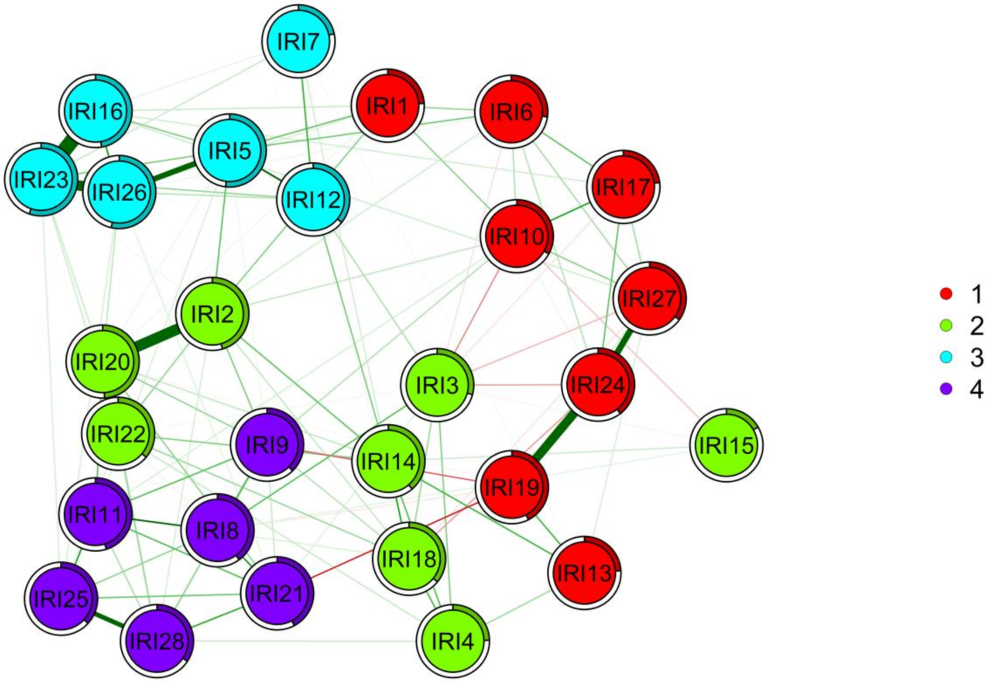

Figure S11. Community detection of IRI items in the low schizotypy group assessed using the "walktrap" algorithm. Edges in green indicate positive partial correlations, while red lines indicate negative ones. The thicker the line, the stronger the connection. The white ring around the nodes shows the amount of variance that item covers. The IRI items were forced into best-fit groups. Low schizotypy settled into 4 groups. IRI: Interpersonal Reactivity Index.

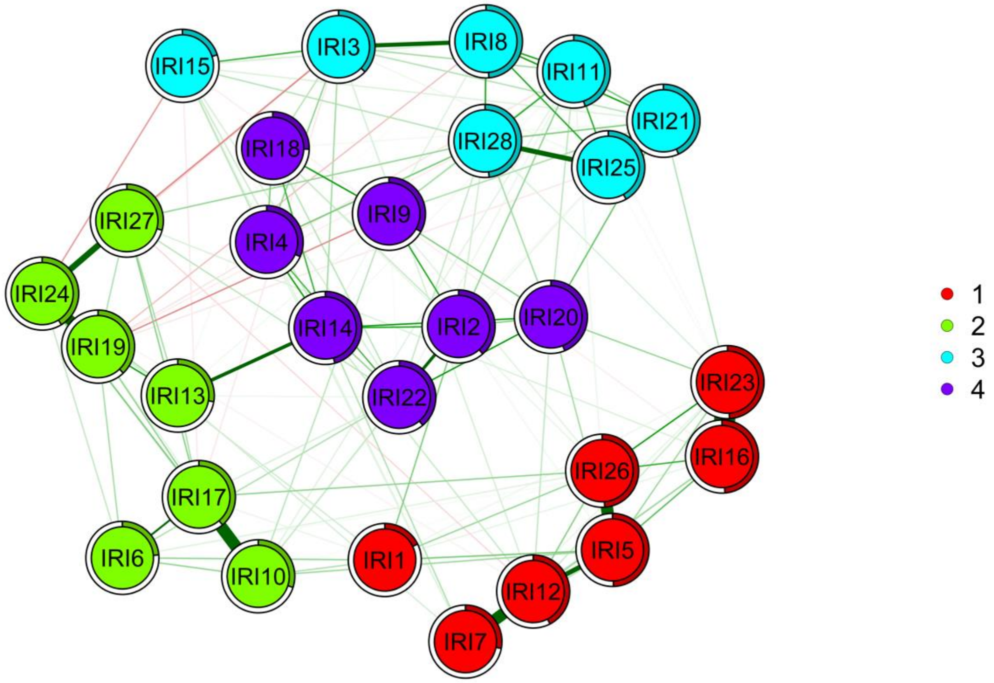

Figure S12. Community detection of IRI items in the high schizotypy group assessed using the "walktrap" algorithm. Edges in green indicate positive partial correlations, while red lines indicate negative ones. The thicker the line, the stronger the connection. The white ring around the nodes shows the amount of variance that item covers. The IRI items were forced into best-fit groups. High schizotypy settled into 4 groups. IRI: Interpersonal Reactivity Index.
